# Transcriptome sequence resource for the cucurbit powdery mildew pathogen *Podosphaera xanthii*

**DOI:** 10.1038/s41597-019-0107-5

**Published:** 2019-06-19

**Authors:** Rita Milvia De Miccolis Angelini, Stefania Pollastro, Palma Rosa Rotondo, Cataldo Laguardia, Domenico Abate, Caterina Rotolo, Francesco Faretra

**Affiliations:** Department of Soil, Plant and Food Sciences, University of Bari, Bari, Italy

**Keywords:** RNA sequencing, Fungal genomics, Fungal genetics

## Abstract

*Podosphaera xanthii* is the main causal agent of cucurbit powdery mildew in Southern Italy. Illumina sequencing of mRNA from two *P. xanthii* isolates of opposite mating types (*MAT1-1* and *MAT1-2*) and their sexual cross was used to obtain a detailed *de novo* Trinity-based assembly of the transcriptome of the fungus. Over 60 million of high-quality paired-end reads were obtained and assembled into 71,095 contigs corresponding to putative transcripts that were functionally annotated. More than 55% of the assembled transcripts (40,221 contigs) had a significant hit in BLASTx search and included sequences related to sexual compatibility and reproduction, as well as several classes of transposable elements and putative mycoviruses. The availability of these new transcriptomic data and investigations on potential source of genetic variation in *P. xanthii* will promote new insights on the pathogen and its interactions with host plants and associated microbiome.

## Abstract (summary)

**Table:** 

Design Type(s)	transcription profiling design • sequence assembly objective • sequence annotation objective • disease analysis objective
Measurement Type(s)	transcription profiling assay
Technology Type(s)	RNA sequencing
Factor Type(s)	strain
Sample Characteristic(s)	Podosphaera xanthii

Machine-accessible metadata file describing the reported data (ISA-Tab format)

## Background & Summary

Cucurbit powdery mildew (CPM) is a common and severe disease of cucurbit crops, producing a characteristic white powdery fungal growth on leaves, stems, petioles and rarely on fruits which can cover the entire host surface causing heavy crop yield and quality losses in most areas of the world^1–3^. Disease management is not easy and often requires numerous applications of plant protection products, including synthetic fungicides, natural substances and microbial antagonists. Several fungi are reported as causal agents of CPM^4^. In Southern Italy, as well as in other geographic areas, the main pathogen responsible for the disease is the ascomycete *Podosphaera xanthii*, an ectophytic and biotrophic pathogen. The fungus has the potential to evolve and differentiate new more adapted genotypes that can overcome genetic resistance of crop varieties and efficacy of new fungicides. Sexual reproduction represents an important source of genetic variation in pathogen populations. *P. xanthii* shows a heterothallic bipolar mating system with both mating types (*MAT1-1* and *MAT1-2*) detected in fungal populations occurring in both field and greenhouse crops in South Italy^5^.

The genetic structure within *P. xanthii* populations has been investigated using molecular markers and by analysing specific genes or functional gene categories such as those responsible for pathogenicity or fungicide resistance^6,7^. Transcriptomic sequences from a single isolate of *P. xanthii* during its ectophytic growth on the host leaf surface has been used to identify secreted proteins with a putative role in pathogenesis^8^ and characterize some of them through host-induced gene silencing (HIGS) mediated by *Agrobacterium tumefaciens*^9^.

Additional sources of variation in fungi are transposable elements (TEs) and cytoplasmic genetic materials, also including mycoviruses, *i.e*. viruses infecting fungi. TEs are ‘jumping’ DNA sequences, moving from one location to another of the genome, recognised as extraordinary contributors to genomic variation and evolution in most eukaryotes and prokaryotes^10^. They play a role also in host-pathogen interactions since effector genes are located within or in proximity to TE-rich genomic regions of pathogens^11^. The role of TEs in *P. xanthii* remains to be investigated. Most mycoviruses do not have visible effects on their hosts but some of them cause debilitation or reduced virulence and have the potential to be developed as innovative biocontrol agents^12^. An increasing number of viral genomes from different fungal pathogens have been recently sequenced and deposited in public databases but there are no records of mycoviruses from *P. xanthii* until now.

We report here Illumina sequencing and *de novo* assembly of the transcriptome from two *P. xanthii* isolates of opposite mating type and their sexual cross aimed at obtaining a more comprehensive transcriptome and improving the resources for investigations on interactions among *P. xanthii*, host plants and the associate microbiome.

## Methods

**Fig. 1:**
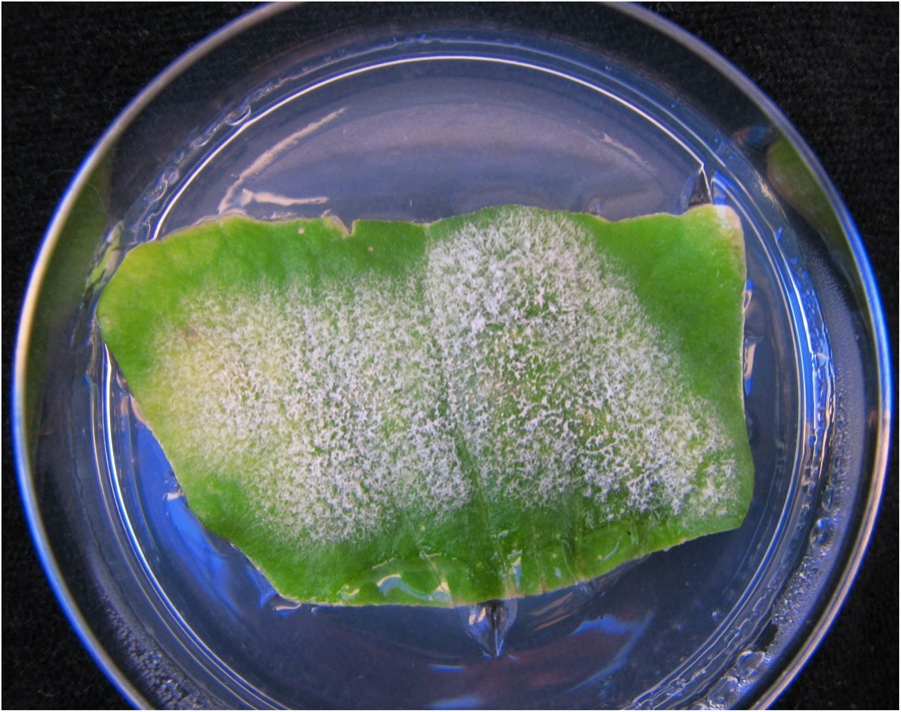
Pairing between the two strains of opposite mating type, G24 (*MAT1-1*) and 7A (*MAT1-2*), after 15 days of growing on zucchini cotyledons.

The *MAT1-1* reference strain G24 was kindly supplied by Prof. M.T. McGrath (Cornell University, USA) while the *MAT1-2* strain 7A was isolated from *Cucurbita pepo* cv. Roberta in Apulia region, South Italy in 2014. Both strains are maintained in the fungal collection at the Plant Pathology Section of the Department of Soil, Plant and Food Sciences of University of Bari and are freely available upon request, without any restriction. Growing conditions on zucchini cotyledons were as described by Miazzi *et al*.^3^. For mating, the two strains were paired on single cotyledons (5 mm apart) and grown for 15 days (Fig. 1). Mycelium and conidia of each strain and their pairing were scraped from the surface of infected cotyledons, and total RNA was extracted using TRI Reagent (Sigma-Aldrich, Milan, Italy) according to the manufacturer’s protocol. cDNA libraries of a 400-bp average-sized fragments were obtained using TruSeq RNA Sample Preparation Kit v2 (Illumina, Inc., San Diego, CA, USA) and sequenced (Illumina Sequencing Technology; HiScanSQ platform; SELGE Network Sequencing Service) to obtain a total of 5.5 Gb corresponding to 59.53 M reads (92-bp paired-end reads; QS ≥ 30)^13^ (Table 1). Reads were analysed for quality statistics, nucleotide distribution and redundancy using FastQC^14^ and trimmed to discard low-quality reads (less than 2%) with Trimmomatic^15^. Raw reads were aligned against the *Cucurbita pepo* reference genome v.3.2 using the CLC Genomics Workbench (CLC bio, Aarhus, Denmark) to filter out contaminant sequences from zucchini cotyledons on which *P. xanthii* strains were grown (1.8 to 6.6% reads for the three libraries). Trinity software was used for *de novo* assembly of the transcriptome using sequencing data from the three libraries^16^. To reduce redundancy, the assembled sequences were then merged and reassembled by using CAP3 software with a minimum overlap length of 50 and at least 95% identity^17^. After CAP3 clustering, the obtained total contigs (71,095), corresponding to putative transcripts and including isoforms and unigenes (54,561)^18^, were functionally annotated using local BLAST+^19^ and Blast2GO PRO to predict Gene Ontology (GO) terms, to assign the assembled sequences to the Kyoto Encyclopedia of Genes and Genomes (KEGG) pathways, and to analyse protein domains using the InterProScan tool^20^.

**Table 1:** List of raw reads.

Organism	Sample	Protocol 1	Protocol 2	Protocol 3	Read-pairs	Biosample	SRA Data Accession
*Podosphaera xanthii*	G24 (*MAT1-1* strain)	Collection of mycelium and conidia grown on zucchini cotyledons	RNA extraction	RNA-Sequencing (paired-end)	10,290,621	SAMN10435485	SRP169883 (SRR8216439)
*Podosphaera xanthii*	7A (*MAT1-2* strain)	Collection of mycelium and conidia grown on zucchini cotyledons	RNA extraction	RNA-Sequencing (paired-end)	10,014,726	SAMN10436485	SRP169883 (SRR8216440)
*Podosphaera xanthii*	Mating G24 × 7A (*MAT1-1* × *MAT1-2* strains)	Collection of mycelia and conidia of *MAT1-1* and *MAT1-2* strains, paired on zucchini cotyledons and grown for fifteen days	RNA extraction	RNA-Sequencing (paired-end)	9,460,612	SAMN10436438	SRP169883 (SRR8216441)

## Data Records

Data generated in this study are publicly available from the NCBI/GenBank database at Bioproject ID PRJNA505479. All raw sequence data have been deposited in the Sequence Read Archive under the accession number SRP169883^13^ (Table 1). The Transcriptome Shotgun Assembly project have been deposited at DDBJ/EMBL/GenBank under the accession GHEF00000000^18^ (Table 2). The annotation dataset of the total Trinity assembly as well as the annotation of putative *P. xanthii* mycoviral sequences, classified according to their sequence homologies with known mycoviruses have been uploaded to figshare^21^.

**Table 2:** Assembly statistics.

Type	Total assembled contigs^¥^	*P. xanthii* transcriptome^¥^	Available *P. xanthii* transcriptome^¥¥^
Number of transcripts	71,095	23,065	37,241
Number of unigenes	54,561	16,418	Nd
Average transcript length (bp)	1,115	1,934	781
Transcript N50	2,251	3,289	923
Maximum length (bp)	14,865	14,865	5,775
Total assembled bases (Mb)	79.3	44.6	29.1
GC content (%)	42.9	42.9	44.0

^¥^This study^18^.

^¥¥^Transcriptome Shotgun Assembly accession GEUO00000000^8^. Nd: not determined.

## Technical Validation

**Fig. 2:**
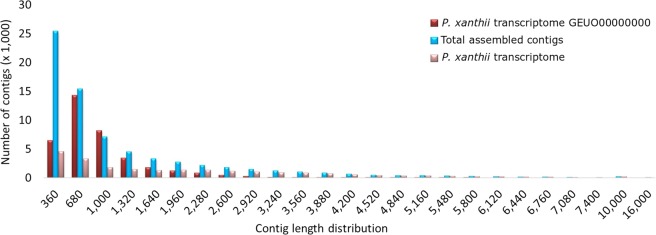
Contig length distribution in the previously available transcriptome of *P. xanthii* including 37,241 transcripts (accession GEUO00000000) as compared to the total assembled contigs (71,095) and the identified transcripts (23,065) of the fungus obtained in this study.

**Fig. 3:**
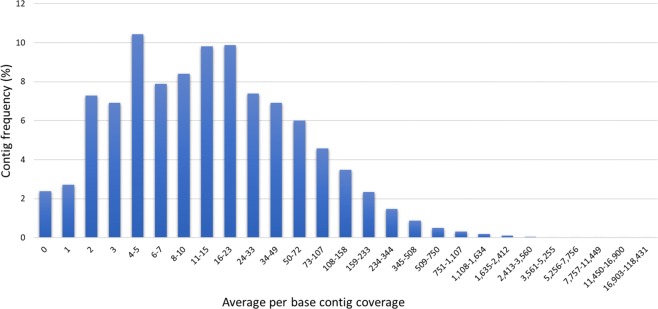
Average per base coverage distribution calculated by alignment of row reads on the *de novo* assembled transcriptome of *P. xanthii*.

**Fig. 4:**
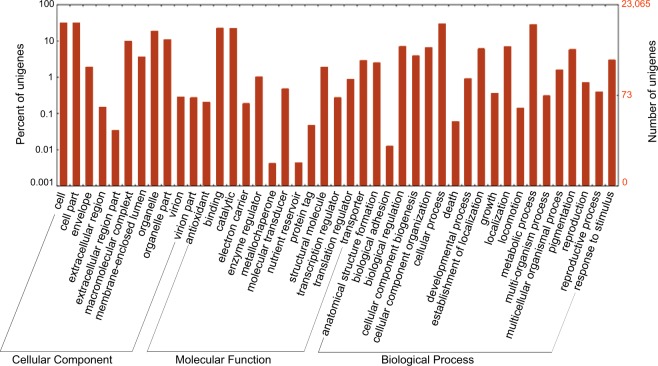
Frequency distribution of Gene Ontology (GO) terms grouped into the main functional categories of cellular component, molecular function and biological process. The right y-axis indicates the number of unigenes per category. The left y-axis indicates the percentage of a specific category of unigenes in the main category.

**Fig. 5:**
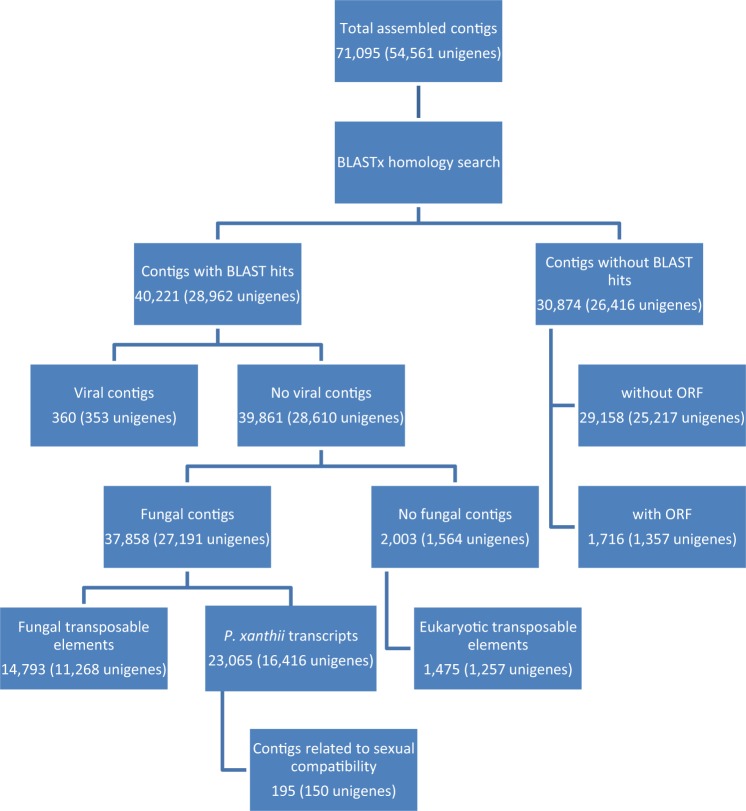
Sets of Trinity-assembled transcripts and related unigenes according to their BLAST annotations.

Our annotated transcriptome draft improves the publicly available *P. xanthii* transcriptome^8^, in terms of completeness and contig size reducing the proportions of fragmented and missing transcripts (Tables 2 and 3 and Fig. 2). The row reads were re-aligned to the *de novo* assembled transcriptome using the CLC Genomics Workbench. Quality control of alignment data was performed with Qualimap 2 to obtain read alignment and coverage statistics^22^ (Fig. 3). Of the total reads, 81.7% successfully mapped in pairs and 14.5% mapped in broken pairs to the assembled transcriptome. A total of 19,324 putative Open Reading Frames (ORFs) were predicted within transcript sequences by TransDecoder and 79.1% were complete. BUSCO^23^ was used to evaluate transcriptome completeness based on a set of 1,438 conserved fungal orthologs, showing that 86% of the assembled transcripts were complete, with 20% of estimated duplication level, and few fragmented (6%) and missing (7%) transcripts (Table 3). The assembled contigs (71,095) were grouped in sequence sets according to their BLASTx annotation^21^. More than 55% of them (40,221 contigs), mapping more than 70% of row reads, had a significant hit in BLASTx search (Table 4). They included a large fraction (95.1%) of sequences showing homology to proteins of Fungi and Oomycetes with the highest similarities with *Erisiphe necator* and *Blumeria graminis* f.sp. *hordei*. Sequences with no significant hits were mostly short fragments or non-coding RNA sequences. A total of 5,013 Gene Ontology (GO) terms, including the three main categories of biological process (3,171), molecular function (1,195) and cellular component (647), were assigned to 24,048 unigenes. WEGO was used to perform functional classification of Trinity unigenes based on the GO annotation^24^ (Fig. 4). Among the identified *P. xanthii* putative transcripts at least 195 sequences related to sexual compatibility and reproduction of the fungus were identified^21^ (Fig. 5). Three hundred sixty contigs showed homology with sequences of viral origin, including several known mycoviruses having double stranded (ds)RNA [*i.e. Totiviridae* (308), *Partitiviridae* (7) or unclassified dsRNA (3)] or positive single stranded + (ss)RNA [*i.e. Narnaviridae* (18), *Ourniavirus* (10)] genomes and unclassified virus-like sequences from fungi (14)^21^ (Fig. 5). They represent novel putative mycoviruses infecting *P. xanthii* that should be further characterized to explore their potential effects on the virulence of the hosting strains. Putative transposable elements (TEs) in the assembled transcriptome were identified and classified by similarity search against Repbase, the reference database of eukaryotic repetitive DNA^25^, by using the CENSOR software tool with default parameters^26^. Overall, 14,793 contigs were homologous to fungal TEs and 1,475 contigs were homologous to TEs identified in other Eukaryotes (Fig. 5). The NonLTR/Tad1 (44.0%) followed by LTR/Gypsy (28.4%), LTR/Copia (17.2%) and DNA/Mariner (8.9%) were the most represented classes among the fungal TEs while LTR/Copia (79.1%) followed by LTR/Gypsy (12.8%) were the most represented among Eukaryotic TEs.

**Table 3:** BUSCO analysis of assembly completeness.

Type	Total assembled contigs^¥^	*P. xanthii* transcriptome^¥^	Available *P. xanthii* transcriptome^¥¥^
BUSCOs complete	1,251 (87%) Total 958 (67%) Single copy 293 (20%) Duplicated	1,242 (86%) Total 954 (66%) Single copy 288 (20%) Duplicated	830 (58%) Total 681 (82%) Single copy 149 (18%) Duplicated
BUSCOs fragmented	87 (6%)	93 (6%)	406 (28%)
BUSCOs missing	100 (7%)	103 (7%)	202 (14%)

^¥^This study^18^.

^¥¥^Transcriptome Shotgun Assembly accession GEUO00000000^8^. Percentages refer to the BUSCO dataset including 1,438 conserved fungal orthologs (http://busco.ezlab.org/v1).

**Table 4:** Annotation statistics.

Type	Numbers
Total transcripts	71,095
No Blast Hits	30,874
With Blast Hits	6,352
With Mapping	16,532
With GO Annotation	17,353
Transcripts with significant hit (%)	40,221 (56.6%)

## ISA-Tab metadata file

Download metadata file

## Data Availability

The following parameters were used to trim row reads with Trimmomatic (version 0.36)^15^: (i) LEADING and TRAILING = 3, removing bases from the two ends of the reads if below a threshold quality of 3; (ii) SLIDING WINDOW = 4:2, cutting the reads when the average quality within the window composed of 4 bases falls below a threshold equal to 2; (iii) MINLEN = 50, removing the reads shorter than 50 bp. CLC Genomics Workbench (version 7.0.3) was used with default parameters for alignment of reads. Default assembly parameters of Trinity (version 2.1.1) were used, with the addition of the “–jaccard_clip” function because a high gene density with overlapping of UnTranslated Region (UTR) was expected (https://github.com/trinityrnaseq/trinityrnaseq/wiki/Running-Trinity). Local BLAST+(version 2.3.0)^19^ was used for BLASTx search against the NCBI non-redundant protein database (downloaded 10 January 2018) setting E-value cut off at 10^−3^. TransDecoder (version 2.1, http://transdecoder.github.io) and BUSCO (version 1.2)^23^ were used with default parameters.
